# Mechanisms Involved in Childhood Obesity-Related Bone Fragility

**DOI:** 10.3389/fendo.2019.00269

**Published:** 2019-05-03

**Authors:** Maria Felicia Faienza, Gabriele D'Amato, Mariangela Chiarito, Graziana Colaianni, Silvia Colucci, Maria Grano, Filomena Corbo, Giacomina Brunetti

**Affiliations:** ^1^Department of Biomedical Sciences and Human Oncology, University of Bari Aldo Moro, Bari, Italy; ^2^Neonatal Intensive Care Unit, Di Venere Hospital, Bari, Italy; ^3^Department of Emergency and Organ Transplantation, Section of Human Anatomy and Histology, University of Bari, Bari, Italy; ^4^Department of Basic and Medical Sciences, Neurosciences and Sense Organs, Section of Human Anatomy and Histology, University of Bari Aldo Moro, Bari, Italy; ^5^Department of Pharmacy-Drug Science, University of Bari Aldo Moro, Bari, Italy

**Keywords:** osteoporosis, low grade inflammation, osteoimmunology, osteoclast, cytokines

## Abstract

Childhood obesity is one of the major health problems in western countries. The excessive accumulation of adipose tissue causes inflammation, oxidative stress, apoptosis, and mitochondrial dysfunctions. Thus, obesity leads to the development of severe co-morbidities including type 2 diabetes mellitus, liver steatosis, cardiovascular, and neurodegenerative diseases which can develop early in life. Furthermore, obese children have low bone mineral density and a greater risk of osteoporosis and fractures. The knowledge about the interplay bone tissue and between adipose is still growing, although recent findings suggest that adipose tissue activity on bone can be fat-depot specific. Obesity is associated to a low-grade inflammation that alters the expression of adiponectin, leptin, IL-6, Monocyte Chemotactic Protein 1 (MCP1), TRAIL, LIGHT/TNFSF14, OPG, and TNFα. These molecules can affect bone metabolism, thus resulting in osteoporosis. The purpose of this review was to deepen the cellular mechanisms by which obesity may facilitate osteoporosis and bone fractures.

## Introduction

Childhood obesity represents an international public health problem with epidemic proportions (1). The World Obesity Federation showed a strong increase of childhood overweight and obesity in several low-, middle-, and high-income regions over the past three decades (2). In the USA ~17% of children and adolescents are obese, representing a risk for health status in adulthood and life expectancy (3, 4).

**Figure 1:**
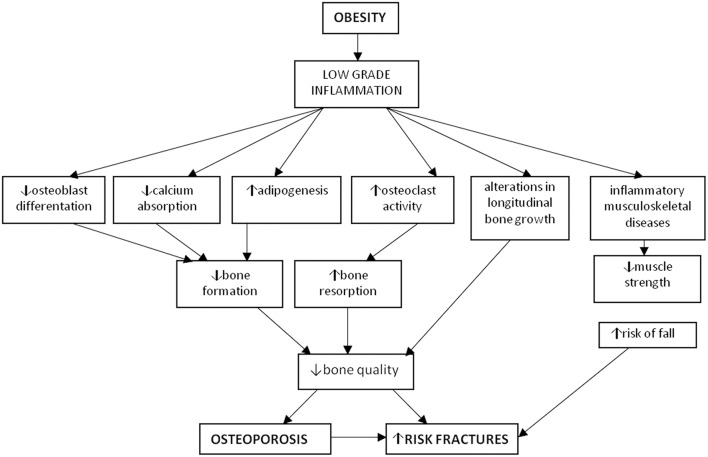
Osteoporosis and obesity. Obesity is characterized by a low-grade chronic inflammation leading to increased osteoclastogenesis and adipogenesis, together with decreased osteoblastogenesis and muscle strength thus determining osteoporosis and increased risk of fractures.

The excess of adipose tissue causes inflammation, oxidative stress, apoptosis and mitochondrial dysfunctions (5, 6). Therefore, obesity can lead to the onset of type-2-diabetes, liver steatosis, cardiovascular and neurodegenerative diseases which can develop early in life (7–12). Different studies have shown a susceptibility to skeletal fractures in obese children (13–23), suggesting that adipose tissue affects bone metabolism (24, 25). Therefore, the excess of fat could reduce the peak of bone mass reached during childhood and adolescence, with a potential osteoporotic risk in adulthood (1, 26). The bone fragility in obese population is due to an increase in fall injury risk, an unbalanced diet and a low physical activity. Despite the knowledge about the interplay between bone and adipose tissue is still growing, recent findings suggest that the influence of adipose tissue on bone can be fat-depot specific. In fact, the visceral fat storage may determine negative consequences on bone, while normal fat depots seem to affect positively the skeleton (27). Furthermore, obesity can act in a different way on specific skeletal compartments (i.e., trabecular vs. cortical) and sites (i.e., weight-bearing vs. non-weight-bearing) (28). The aim of our review was to overview the cellular mechanisms by which obesity regulates bone remodeling leading to osteoporosis and fracture risk (Figure 1).

## Mesenchymal Stem Cell Fate

The link between obesity and osteoporosis can be explained by the common stem cell precursor shared by osteoblasts and adipocytes (29). Two groups of crucial factors, CEBP-α, -β, - δ, and PPAR-α, -γ2 and -δ, need to be activated to attain a complete adipocytic differentiation of a mesenchymal stem cell. Otherwise, activation of other crucial factors (i.e., RUNX2, BMP2, TGF-β, and Osterix) are required to shift the differentiation of a mesenchymal cell into osteoblast (29). The differentiation “switches” characterizing stem cell fate are strictly linked to the stimuli present in the microenvironment. Furthermore, adipocytes cultured from marrow display the capability to revert to a proliferative status and thus differentiate in osteoblasts (30).

## Obesity and Bone Turnover

The link between obesity and bone turnover has been evaluated both in humans and murine models, and the excess of fat mass is associated with reduced bone mineral density (BMD) (31–34). Obesity influences bone metabolism by different mechanisms. It stimulates pre-osteoblasts to differentiate toward adipocytes rather than osteoblasts, thus filling the cavities of bone marrow with adipocytes rather than trabecular bone with consequent bone fragility increase (35). Consistently, in obese adolescents and young adults, total and trabecular BMD and trabecular number have been inversely related with marrow adipose tissue (MAT) at the distal tibia, but not with lumbar spine MAT (36). Obesity can also enhance bone resorption by the increase of pro-inflammatory cytokine levels [Tumor Necrosis factor alpha (TNFα) and interleukin-6 (IL-6)], which promote osteoclast formation and activity by affecting RANKL/RANK/OPG pathway (37, 38). Bone marrow fat also may regulate osteoclastogenesis by producing RANKL (39). Obese subjects show low serum levels of adiponectin (40), an adipokine that inhibits osteoclast formation and activity (41). High leptin levels associated with reduced adiponectin may stimulate both macrophage accumulation into the adipose tissue (42) and adhesion of macrophages to endothelial cells (43). Several studies have demonstrated the impact of obesity on bone remodeling. Weiler et al. found that body fat percentage is correlated with suboptimal achievement of peak of bone mass in a cross-sectional study involving 60 girls (10–19 years old) (44). Goulding et al. showed that severe obesity is associated with higher risk of distal forearm fractures in boys aged 3–19 years (16).

Furthermore, Hsu et al. reported an increased risk for osteoporosis and non-spine fractures related with high percentage of body fat in a cross-sectional study involving 7,137 men, 2,248 postmenopausal women and 4,585 premenopausal women aged 25–64 years old (33). In leptin-deficient (ob/ob) obese mice, a reduction of femoral BMD, trabecular bone volume, and cortical thickness has been observed (45). Using a mouse model of diet-induced obesity, it has been found that mice fed with a high fat diet (HFD) had cancellous bone loss in the proximal tibia, together with a significant body weight increase (46). In the models, an increase of leptin and TRAP serum levels, a high RANKL/OPG ratio in cultured osteoblasts, and in the number of osteoclasts was observed (46–48). HFD determines an augment of bone marrow adiposity together with a reduction of BMD in several bone segments, and an increase of IL-6, TNFα, peroxisome proliferator-activated receptor γ (PPARγ) (49). Additionally, HFD decreases intestinal absorption of calcium, through the production of unabsorbable calcium soaps by free fatty acids (50–52).

## High Levels of Pro-inflammatory Cytokines in Obesity

**Figure 2:**
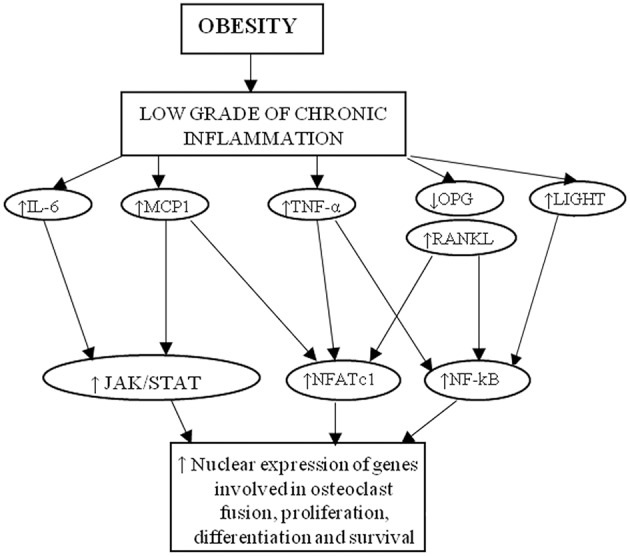
Cytokines linking obesity to osteoporosis. MCP-1, RANKL, IL-6, TNFα, and LIGHT activate intracellular pathway that induce the nuclear expression of genes involved in osteoclast formation, activity and survival.

Obesity is characterized by a low-grade chronic inflammation. The discovery of high TNFα levels in the adipose tissue of obese mice offered the initial demonstration of a cross-talk between obesity and inflammation (27). Furthermore, the detection of leptin, hormone produced by adipocytes, further sustained the idea that adipose tissue is not only an energy storage but it is also a dynamic endocrine organ (53, 54). In fact, the chronic inflammatory status associated to obesity is characterized by abnormal cytokine production, and activation of signaling pathways of inflammation, with consequent development of obesity-related diseases (55). Adipose tissue is rich of macrophages, key source of inflammatory cytokines (56, 57). Obese subjects produce higher amounts of TNFα and pro-inflammatory cytokines (IL-6 and C-reactive protein) in adipose tissue than lean controls (58–60). Furthermore, the levels of adiponectin are lower in obese patients respect to controls (40). Obesity has also been related with inflammatory musculoskeletal diseases (i.e., osteoarthritis) (61). The low-grade inflammation which characterizes obesity may also influence endochondral longitudinal bone growth together with change in nutrients, minerals, and hormone metabolism (62). In obesity, the altered levels of numerous molecules inhibit osteoblastogenesis, as TNFα, DKK1, sclerostin, IL-6, serotonin, and advanced glycation end products (AGEs) [revised in Roy et al. (63)]. Interestingly, many pro-inflammatory cytokines involved in obesity are also crucial players of osteoclast formation and activation, and are known to be involved in bone disease (64–66), suggesting a link between obesity and bone turnover. In particular, in this review we focused the attention on MCP1, TRAIL, LIGHT, OPG, and TNFα (Figure 2).

## MCP1

The chemokine Monocyte Chemotactic Protein-1 (MCP1) interacts with the receptor CCR2, its expression is ubiquitous and it is up-regulated by numerous stimuli. Firstly, MCP1 has been purified from myelomonocytic cell line THP-1 (67), but it is expressed by numerous normal cells, such as endothelial cells (68–72), fibroblasts (73, 74), mononuclear cells (73, 75–82), mast cells (83), epithelial cells (84), keratinocytes (85), melanocytes (86), smooth muscle cells (68, 87, 88), mesothelial cells (89), adipocytes (90, 91), mesangial cells (92–96), chondrocytes (97), osteoblasts, astrocytes (98, 99), and microglia (99). In untreated normal cells, MCP1 levels are low, while tumor cell lines produce MCP1 constitutively (67, 100–104). The expression of MCP1 can also be downregulated by glucocorticoids (e.g., dexamethasone), cytokines (e.g., IL-13), and nitric oxide (79, 80, 97, 105–109). The expression of MCP1 and its receptor is higher in subcutaneous and visceral adipose tissues of obese patients than controls (90). Additionally, in omental fat of subjects with severe obesity, an increase of MCP1 expression together with an elevated macrophage infiltration was found (91). MCP1 levels are higher in obese adults (110) and children (111) compared to aged-matched controls. In obese patients MCP1 levels were augmented by fructose expenditure (112), reduced by low-glycemic index diet (113), and modulated by PTH (114). Moreover, 1α-25-dihydroxy-vitamin D decreases MCP1 production by adipocytes (115). CCR2-deficient mice fed with a HFD showed insulin resistance and reduced accumulation of visceral fat (78). Furthermore, MCP1 exerts a pro-angiogenic action (116), thus contributing to the expansion of adipose tissues.

MCP1 interaction with CCR2 on monocytes/macrophages leads to osteoclastogenesis via JAK/STAT and Ras/MAPK signaling pathways. However, RANKL co-treatment is mandatory to generate active bone resorptive osteoclasts (117).

## TRAIL

TRAIL is a TNF superfamily member, initially known for its selective pro-apoptotic activity on cancer cell death (118). In humans, TRAIL binds to its death domain (DD)-containing receptors, DR5 and DR4, as well as decoy receptors osteoprotegerin (OPG), DcR1 and DcR2. In contrast to humans, mice express only one death receptor, mDR5, showing about 60% sequence homology to human DR4 and DR5 (118), and as decoy receptors, mDcR1, mDcR2 and OPG. TRAIL also affects non-cancer cell viability and activity, such as thymocytes (119), neural cells (120), hepatocytes (121), osteoclasts (122, 123), stem cells (124), valvular interstitial cells (125, 126), vascular smooth muscle cells (127), and osteoblasts (128–130). TRAIL pro-apoptotic signal in undifferentiated osteoblasts determines the activation of caspases (131). In lymphomonocyte cultures from donors TRAIL directly induces osteoclastogenesis in the absence of RANKL, whereas generates an inhibitory action when used simultaneously to RANKL (132). This last condition is associated with the inhibition of the phosphorylation of P38/MAPK (133). TRAIL controls homeostasis of the immune system in health and disease. Zoller et al. demonstrated that TRAIL determines an inflammatory status in pre-adipocytes and adipocytes (134). Funcke et al. reported that TRAIL induces the proliferation of human pre-adipocyte via ERK1/2 activation (135). Consistently, TRAIL takes part in the pathogenesis of metabolic diseases, i.e., obesity (121, 136). It has been demonstrated a positive association between TRAIL serum levels and body fat, serum lipid concentrations (137), waist-circumference and fat mass in healthy subjects (138). TRAIL serum levels were also positively correlated with higher energy balance (139), LDL and waist circumference, supporting a significant link between visceral adiposity and TRAIL (140). Even if these reports demonstrated high TRAIL levels in obesity, other authors failed to show such correlation (125, 138, 141, 142). Furthermore, a positive correlation between weight gain and TRAIL has been demonstrated in obese animal models. In detail, in adipose tissues of leptin-deficient mice the expression of TRAIL was significantly higher respect to wild-type mice (125). Furthermore, TRAIL levels decreased following an overnight fasting, and then rescued following feeding (125). Otherwise, results derived from TRAIL-treated wild-type and HFD fed, or TRAIL-deficient mice, support a defensive role for TRAIL in obesity. Bernardi et al. reported that in mice fed with a HFD, weekly injections of TRAIL generated a smaller fat mass compared to controls. TRAIL-mediated weight loss was linked to decreased transcript levels of TNFα, caspase-3, MCP1, augmented apoptosis in adipocytes, and decreased IL-6 serum levels (143). Consistently, TRAIL^−/−^ApoE^−/−^ mice fed with HFD showed high levels of IL-6 and MCP1, together with adipocyte hypertrophy and weight gain respect to ApoE^−/−^ mice (144). Although Di Bartolo et al. (144) and Bernardi et al. (143) suggest that TRAIL may be beneficial to treat obesity, conversely Keuper et al. (125) found that TRAIL stimulated *in vitro* insulin resistance in adipocytes. Thus, considering the effect of TRAIL on adipose tissue together with its pro-osteoclastogenic and osteoblastic pro-apoptotic effects, further studies are needed to elucidate the role of TRAIL in obesity and related bone disease, overall in childhood.

## LIGHT/TNFSF14

LIGHT (homologous to Lymphotoxins exhibiting Inducible expression and competing with herpes simplex virus Glycoprotein D for herpes virus entry mediator [HVEM], a receptor expressed by T-lymphocytes) is part of TNF superfamily (TNFSF14) and a crucial cytokine of the TNF-lymphotoxin network (145–148). It is expressed by natural-killer cells, activated T-cells, granulocytes, monocytes, and immature dendritic cells (149–151). LIGHT can bind two receptors, lymphotoxin-beta receptor (LTβR) and Herpes virus entry mediator (HVEM). LTβR is present on stromal and myeloid cells (146), HVEM on hematopoietic, epithelial and endothelial cells (151, 152). LIGHT-HVEM interaction determines a potent T-cell co-stimulatory effect (153–156). LIGHT-deficient mice showed an impaired activity of CD8+ T-cells and reduced trabecular bone (157–159). LIGHT has a pro-osteoclastogenic effect and we demonstrated that its high levels are linked to bone-disease patients (160–163). LIGHT triggers osteoclastogenesis through the phosphorylation of Akt, nuclear factor-κB (NFκB) and JNK pathways, it indirectly also inhibits osteoblastogenesis through immune cells (160). Moreover, LIGHT is involved in adipogenesis (164, 165). In detail, Tiller et al. reported that LIGHT inhibits adipose differentiation without affecting adipocyte metabolism (166). Otherwise, Kim et al. demonstrated that LIGHT has a key role in adipose tissue inflammatory responses through the increase of macrophages/T-cell infiltration and the release of inflammatory cytokines. In this system LIGHT effect is HVEM-mediated (164). HVEM deficiency displays a protective role against adipose tissue inflammation induced by ovariectomy (165). It has been reported that LIGHT signaling attenuates beige fat biogenesis (167). Human studies demonstrated high LIGHT levels in obese adults compared to controls (168). Interestingly, our preliminary results showed high LIGHT levels in obese children (169).

## OPG/RANKL

Osteoprotegerin (OPG), soluble receptor for TRAIL and RANKL, is part of the TNF receptor superfamily. OPG, primarily known as bone resorption inhibitor, shows also anti-apoptotic and anti-inflammatory effects (170). OPG role has been evaluated in metabolic diseases (171). Indeed, low levels of OPG have been found in non-alcoholic fatty liver disease (NAFLD), important consequence of obesity (172, 173). Erol et al. found that obese children showed significantly lower OPG levels compared to the controls. A reduction of OPG levels in obese subjects has been described in some studies (174, 175), otherwise no relationship has been found between BMI and OPG in other reports (176, 177). Interestingly, Ugur-Altan et al. (174) found that the lowest OPG levels are associated with the highest HOMA-IR values, and serum OPG levels negatively correlated with fasting insulin, HOMA-IR, and glucose. Otherwise, Suliburska et al. (178) showed that obese adolescents displayed higher OPG levels compared to controls, that positively correlated with insulin resistance. Studies on adults reported a potential correlation between metabolic syndrome, insulin resistance, NAFLD, and OPG levels (172–181). These studies demonstrated that in NAFLD the levels of OPG in sera could be utilized as a non-invasive liver damage indicator (174).

Obesity is also associated with increased secretion of RANKL by osteoblasts as well as elevated levels of the RANKL/OPG ratio (182). RANKL-RANK interaction leads to the activation of the transcription factors NFκB and AP-1, which in turn activates nuclear factor of activated T-cells, cytoplasmic 1 (NFATc1). The latter translocates into the nucleus, thus inducing the expression of genes involved in osteoclast formation and activity.

## TNFα

TNFα is a pro-inflammatory molecule involved in the regulation of inflammatory response, cell differentiation, proliferation, and apoptosis (183). TNFα binds two receptors, type 1 or 2, and activates NFkB and MAPK signaling (184), and is produced mainly by stromal–vascular cells and adipose tissue macrophages (185). TNFα is an inhibitor of osteoblastogenesis (186), adipogenesis and adipocyte differentiation, mainly by binding TNFR1 and activating the NFκB, ERK1/2 and JNK pathways (187). Another mechanism by which TNFα inhibits adipogenesis is the activation of Wnt/β-catenin pathway and inhibition of transcription factors, such as PPARγ and C/EBPs (188, 189). High levels of TNFα have been found in obese and diabetic subjects (58). The TNFα treatment in 3T3-L1 cells and rats induces insulin resistance (190), whereas the suppression of TNFα and receptor genes improves insulin sensitivity in ob/ob rodent model (191). Moreover, TNFα upregulates miR-155 and miR-27 by activating the NFκB pathway, thus inhibiting early adipogenic transcription factors, i.e., C/EBPβ and CREB (192, 193). TNFα also down-regulates miR-103 and miR-143, which accelerate adipogenesis (194).

TNFα shows a pro-osteoclastogenic effect that can be direct or indirect. In detail, for the direct mechanism TNFα binds to TNFR1 through NF-κB, JNK and p38 with consequent activation of NFATc1, which promotes the transcription of genes involved in osteoclast formation and activity. Moreover, TNFα indirectly affects osteoclast formation by promoting RANKL expression in bone marrow stromal cells (195). Otherwise, TNFα promoted osteoclastogenesis only in the presence of RANKL permissive levels (196).

## Conclusions

Although childhood obesity has not been yet identified as a direct cause of osteoporosis, several cellular mechanisms linked to the accumulation of fat in the body can contribute to osteoporosis and bone fractures. Low grade chronic inflammation commonly exists in obese populations and the cytokines negatively affect bone health. Obesity positively regulates osteoclasts functioning by up-regulating the production of RANKL, LIGHT, TRAIL, TNFα, MCP1 and inhibiting osteoblastogenesis, thereby accelerating bone resorption. Future investigations on the relationship between cytokines and adipogenesis are expected to lead to the improvement of management strategies for osteoporosis associated to obesity.

## Author Contributions

MF and GB write the review. FC, MG, and SC critically revised the paper. GC, GD, and MC performed the bibliographic research and realized the figures. All the authors critically revised the paper.

### Conflict of Interest Statement

The authors declare that the research was conducted in the absence of any commercial or financial relationships that could be construed as a potential conflict of interest.
